# The Performance of an ML-Based Weigh-in-Motion System in the Context of a Network Arch Bridge Structural Specificity

**DOI:** 10.3390/s25154547

**Published:** 2025-07-22

**Authors:** Dawid Piotrowski, Marcin Jasiński, Artur Nowoświat, Piotr Łaziński, Stefan Pradelok

**Affiliations:** Faculty of Civil Engineering, Silesian University of Technology, ul. Akademicka 5, 44-100 Gliwice, Poland; marcin.jasinski@polsl.pl (M.J.); artur.nowoswiat@polsl.pl (A.N.); piotr.lazinski@polsl.pl (P.Ł.); stefan.pradelok@polsl.pl (S.P.)

**Keywords:** artificial intelligence, machine learning, neural network, network arch bridge, structural health monitoring

## Abstract

Machine learning (ML)-based techniques have received significant attention in various fields of industry and science. In civil and bridge engineering, they can facilitate the identification of specific patterns through the analysis of data acquired from structural health monitoring (SHM) systems. To evaluate the prediction capabilities of ML, this study examines the performance of several ML algorithms in estimating the total weight and location of vehicles on a bridge using strain sensing. A novel framework based on a combined model and data-driven approach is described, consisting of the establishment of the finite element (FE) model, its updating according to load testing results, and data augmentation to facilitate the training of selected physics-informed regression models. The article discusses the design of the Fiber Bragg Grating (FBG) sensor-based Bridge Weigh-in-Motion (BWIM) system, specifically focusing on several supervised regression models of different architectures. The current work proposes the use of the updated FE model to generate training data and evaluate the accuracy of regression models with the possible exclusion of selected input features enabled by the structural specificity of a bridge. The data were sourced from the SHM system installed on a network arch bridge in Wolin, Poland. It confirmed the possibility of establishing the BWIM system based on strain measurements, characterized by a reduced number of sensors and a satisfactory level of accuracy in the estimation of loads, achieved by exploiting the network arch bridge structural specificity.

## 1. Introduction

Structural health monitoring (SHM) comprises a network of interconnected sensors that are essential elements of the entire monitoring framework [1]. Its establishment may require the integration of various sensor types, each allowing the recording and analysis of specific parameters of structural response. One of the widely used types of sensors is fiber optic sensors, which have been successfully applied in many engineering fields, including civil engineering [2,3], geotechnology [4,5], aviation [6], energy [7], and material engineering [8]. They are resistant to corrosion, immune to electromagnetic interference, and versatile in geometric shape [9], and they are also able to detect small variations in measured values [10].

SHM systems have been increasingly used to estimate road [11,12,13,14] and railway traffic load [15,16,17]. This task, known as Weigh-in-Motion (WIM), or Bridge Weigh-In-Motion (BWIM) specifically for bridge-level load recognition, may involve different methods and instrumentation [18]. The strain-based BWIM systems were developed and introduced for various types of railway bridges, including single-span [19], multi-span [20], and truss structures [21]. The use of specific fiber optic sensing technologies was reported by Yoon et al. [22] to analyze the response of a bridge superstructure under a train load, allowing for the identification of car types. Pimentel et al. [23] developed an influence line-based framework to estimate the static axle load, geometry, and speed of trains. Wang et al. [24] proposed the use of crossbeam strain measurement to assess load magnitude using Fiber Bragg Grating (FBG) sensors and the linear superposition algorithm. Although the use of fiber optic sensors in BWIM is apparent, the vast majority of applications involve the measurement of strains in directly loaded components of the railway, including rails, sleepers, and a subgrade [25]. For example, Zhou et al. [26] described the method used to estimate the magnitude and positions of the wheel–rail contact forces using fiber optic sensors mounted on the rails. Mishra et al. [27] designed an FBG-based WIM system to estimate train parameters, including the speed, weight, and axle count of cars. Similar characteristics were analyzed by Martincek et al. [28] who incorporated the interferometric optical fiber sensing, Lan et al. [29] who used the rail- and track slab-embedded sensors, and Mishra et al. [30] who analyzed the impact of train speed and weight on the optomechanical behavior of optical sensors.

In the context of road infrastructure, WIM proposals should address the issues associated with the non-deterministic nature, uncertain positioning, and variety of road traffic scenarios. Reported applications include systems based on the measurement of strains by sensors embedded in pavement [31,32,33,34] and subgrade layers [35]. Several studies documented the implementation of strain gauges in bridge-level WIM, especially for vehicle detection and classification [36], load magnitude estimation [37], and axle count obtainment [38]. Zhao et al. [39] proposed the framework for axle detection using strain measurement and wavelet transformations. Tan et al. [40] described the detection of vehicle configuration and speed using a strain-based BWIM approach enhanced of the regularization and iterative approach. Similar systems were evaluated considering thermal field effects [41] and the spatial variability of structural properties [42]. Heinen et al. [43] examined single-span bridge structures using shear force-based BWIM systems. Among various applications, the use of fiber optic sensing has also been reported. Alamandala et al. [44] proposed a BWIM system using Fiber Bragg Grating (FBG) sensors arranged at different angles. The authors measured wavelength shifts to produce a temporal response curve and predict vehicle parameters, including load and speed. Zhang et al. [45] described a method for assessing vehicle weight considering random traffic flow and using the advantages of long-gauge FBG sensors. Chaudhary et al. [46] proposed an influence line-free BWIM method based on spatiotemporal strain data from distributed fiber optic sensors (DFOS). The gross weight of the vehicles was identified by spline regression and structural mechanics relationships. Oskoui et al. [47] developed an FBG-based sensor to measure end rotations of a bridge girder for the purpose of estimating vehicle load.

Numerous studies describe the issues of optimizing the sensor layout to facilitate decisions about the number and location of sensors and to obtain the most adequate information on the actual behavior of a structure [48]. Several works focused on the analysis of sensor types, power consumption, and efficiency in data acquisition and transmission for different bridge structures [49]. Among various implementations, the use of automated optimization techniques was reported, including genetic algorithm (GA) [50], particle swarm optimization (PSO) [51], and bioinspired methods [52,53,54]. Capellari et al. [55] proposed a Bayesian experimental design approach to optimize sensor layout, including minimizing the number of sensors, selecting their type, and determining their spatial deployment. Gonen et al. [56] described a framework for determining optimal sensor locations for the purpose of identifying modal properties, incorporating uncertainties in modal displacements and hierarchical clustering. Various other applications include the optimal sensor layout design for anomaly detection [57], damage detection [58], and damage identification [59,60]. The current study focuses on recognizing the impact of the structural specificity of a bridge on the potential layout of FBG sensors and accuracy of the BWIM application. Based on the example of a network arch bridge, it is assumed that the number and location of FBG sensors can be reduced while maintaining the performance and satisfactory accuracy of vehicle weight estimation by exploiting structural specifics of the bridge. The network arch, characterized by inclined hangers that cross each other at least twice, allows a significant reduction in the bending moments for both the arch ribs and the girders, a limitation of the load influence surface range, and a general improvement in structural efficacy compared to other types of arch bridges [61]. However, the difficulties in the interpretation of complex traffic conditions with conventional methods have led to an increasing interest in machine learning (ML)-based solutions.

Recent advances in computer science have significantly contributed to the enhancement of SHM systems through the integration of ML-based algorithms [62]. These techniques may be used in many areas of civil engineering, including damage detection [63,64], estimation of structural deformation [65], and dynamic analysis [66]. ML algorithms are also used in the BWIM approach, and their implementation can involve various tools and frameworks. Le et al. [67] proposed the classification approach to detect the structural overload in the railway bridge. Bosso et al. [68] described the regression tree approach to predict vehicle weight and identify the overload trucks. Except for traditional supervised learning, classification, and regression models, transfer learning is also often used with very good results. Yan et al. [69] proposed the transfer learning-enhanced CNN algorithm to identify the gross weight and axle weight of the vehicles passing the bridge. Their research also showed the possibility of reducing the training set using transfer learning. Computer vision is also often used in BWIM systems. Jian et al. [70] proposed a BWIM method with a focus on complicated traffic scenarios. Their method can identify strain influence surfaces of the bridge structure and vehicle weights.

The current research integrates the advantages of FBG sensors and ML algorithms for the purpose of SHM system deployment using a network arch bridge in Wolin, Poland, as an example. A novel combined model- and data-driven framework is proposed consisting of the establishment of the FE model, its validation and adjustment based on static and dynamic load testing, and data augmentation to facilitate the training of selected physics-informed regression ML models. The work analyzes the accuracy and performance of selected ML regression algorithms, namely linear regression (LR), random forest (RF), and neural networks (NN), showing the impact of the structural specificity of the network arch bridge on the performance of the proposed BWIM system.

The current research is structured as follows. Section 2 presents the bridge and SHM system overview, including details on the arrangement and characteristics of the installed FBG sensors, a description of the network arch-specific strain distribution, and the preparation of training data for the purpose of ML-based BWIM deployment. Section 3 discusses the performance and accuracy of selected ML algorithms in predicting vehicle weight and position for various sets of features. Section 4 contains conclusions, limitations, and perspectives of the developed framework.

## 2. Methodology

### 2.1. Bridge and SHM System Overview

The BWIM system, which involves weighing and locating vehicles passing through the structure, was implemented on a steel bridge in Wolin, Poland. The structure is a single-span network arch bridge with a span of 165.0 m. The superstructure consists of two steel arches with a closed box cross section of 1.00 × 1.80 m, is connected by steel circular tube bracings with a diameter of 1.22 m, and is slightly inclined inward in relation to the vertical plane. The bridge deck was suspended to the arch ribs with inclined wire hangers arranged in a network layout. The deck has a form of a steel greed structure composed of two longitudinal tie beams and transverse crossbeams placed at a regular spacing of 6.00 m. The distance between the end and the closest middle crossbeams is set at 7.50 m. The cross section of the tie beams is a closed rectangular box measuring 0.75 m in width and 1.30 m in height. The axial spacing between the tie-beams, measured in the transverse cross section, is 16.15 m. The middle crossbeams have an I section of 0.45 m wide and a variable height ranging from 0.94 to 1.19 m. The end crossbeams are designed as closed box sections that are 2.58 m wide and vary in height from 0.88 to 1.20 m. The steel grid is integrally connected to a concrete deck slab, thus forming a composite superstructure. The slab consists of precast concrete slab members interconnected with a 14.0 cm cast in-place layer. The entire steel structure was made of S460N steel, while the bridge slab was made of C50/60 and C40/50 concrete, for precast and in situ layers, respectively.

To estimate the weight and position of the vehicles on the bridge, an SHM system was installed, consisting of optical sensors based on Giber Bragg Grating (FBG) technology. Three measuring gates were designated on the bridge, located at 1/4, 1/2, and 3/4 of the span, in which a total of 17 measuring points were distinguished, based on the response characteristics of the network structure. Each of the measuring gates consists of three points installed on the crossbeam (CB) and two points installed on the hangers (H), one located on the left and the other on the right hanger. The middle gate includes two additional measurement points in the tie-beams (TB), each on one side of the deck.

**Figure 1:**
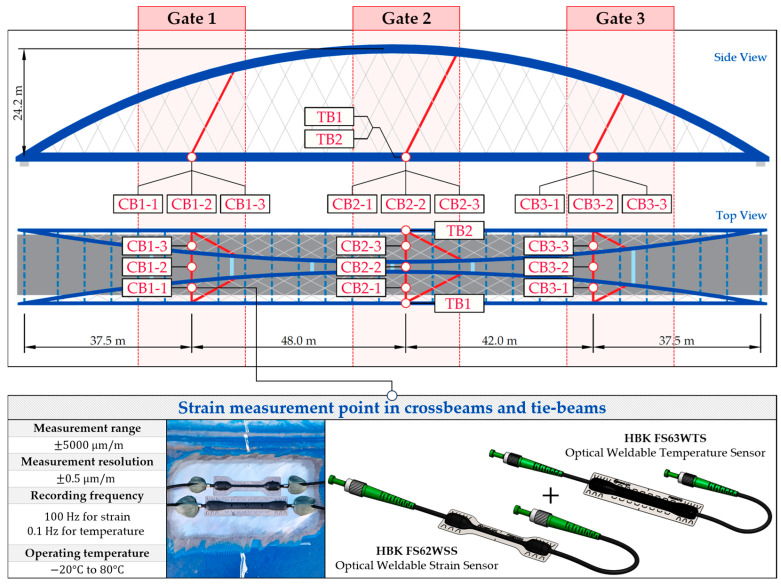
The measurement gates, arrangement, and characteristics of the welded FBG sensors in the crossbeams (CB) and tie-beams (TB).

In the case of the crossbeams and tie-beams, the sensors are spot welded to the structure after the prior cleaning and grinding of the surface. In addition to strain sensors, temperature sensors are also installed near the strain measurement points for temperature compensation purposes. The crossbeam sensors are installed on the top surface of the bottom flanges, whereas those on the tie-beams are mounted on the underside of the section. Subsequently, they were protected against corrosion according to the HBK specification [71], including a high adhesion nonhardening masking sealant AK22, 0.05 mm thick aluminum foil coating, and a 3 mm thick layer of kneadable putty (ABM75). The arrangement and characteristics of the welded strain and temperature sensors in the elements of the deck grid are shown in Figure 1.

**Figure 2:**
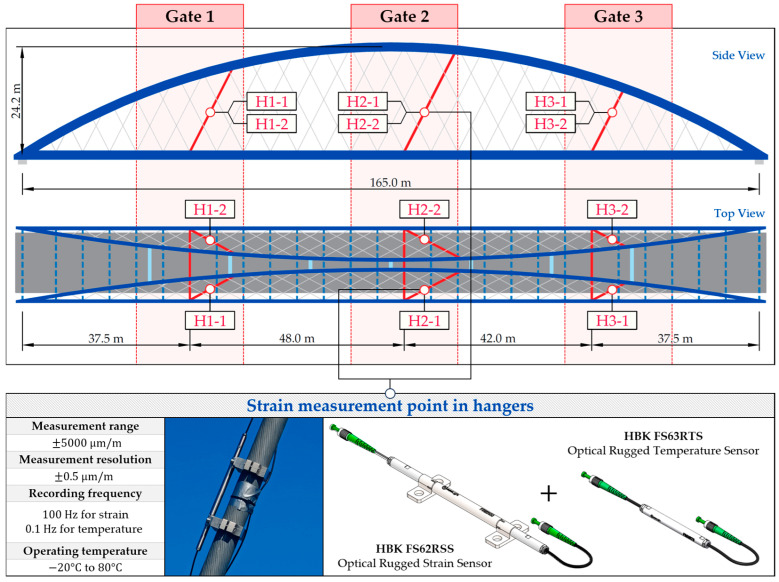
The measurement gates, arrangement, and characteristics of the bolted FBG sensors in the hangers (H).

The hangers installed on the bridge are part of the full locked coil rope system by Teufelberger-Redaelli [72]. These include wire cables of high strength steel that preclude the use of welded strain measurement. Due to the characteristics of the hanger cross-section and the potential torsional effects that may arise in response to the change in tension force, dedicated clamps were fabricated and used for the installation of the strain sensors. They are arranged together with temperature measurement sensors that are attached to the surface of the hanger (Figure 2).

All sensors in the three gates are connected to the breakout cables. These cables extend along the bridge and transmit signals to an MXFS interrogator, with 8 inputs of 16 channels each. The interrogator is installed in a cabinet mounted on one of the bridge end supports. The cabinet is also equipped with an industrial computer for data acquisition, processing, and management, a permanent Internet connection, and an uninterruptible power supply (UPS), which ensures continuous operation in the event of a power outage. The system enables synchronous acquisition from all channels, allowing for time-correlated analysis managed by the Catman Easy v5.6.1 software. Using the software, it is possible to record in real time the strain values at key points of the structure that serve as input data for ML-based BWIM algorithms.

### 2.2. Load Testing and Physics-Informed BWIM Design

After the installation of the sensor system and prior to training of the ML algorithms, load tests were carried out to verify the correct behavior of the structure. Load tests can be an element of the bridge acceptance procedure that confirms design assumptions and allows the estimation of the influence of additional factors on the stiffness and response of the structure. Finally, data obtained on this basis allow for the verification of the SHM system and calibration of the analytical finite element (FE) model, which will ultimately become a source of training data for the purpose of the ML-based BWIM.

The load testing procedure consists of static and dynamic tests. Five different static load schemes were assumed to generate maximum strain values at the selected measurement points. They include three symmetric schemes, one above each of the measurement gates, and two additional asymmetric cases to verify the transverse distribution of loads and the torsional stiffness of the span. The schemes consisted of nine trucks, each of them weighing between 27.8 and 41.5 t, that were introduced onto the bridge to yield the strain at a level of at least 60% of the theoretical strain values caused by the live load model assumed in the design. According to the established procedure, the load is kept on the bridge for a minimum duration of 30 min. The duration of the test is extended by successive 10 min intervals if strain stabilization has not been achieved. Stabilization is defined as no increase in strain values exceeding 2% between consecutive readings within the 10 min measurement interval. The response of the structure is also measured after unloading to observe residual strains and to assess the general ability of the structure to return to its initial state.

The data recorded by the SHM system were used to calibrate the numerical FE model, which was then used to generate a synthetic training set for the ML algorithms. Acquiring an appropriate data set solely through experimental studies would entail significant time and financial expenditure, which is why an approach using simulation data was adopted. The use of the FE model in the procedure allowed us to increase the variety of loads examined in relatively short time compared to the site experiments. It enabled the analysis of vehicular loads of varying magnitude, axle configuration, and placement, among which single- and multi-vehicle scenarios of different structure can be introduced. In addition to the increase in the size and diversity of datasets, the FE model enables analyzing the response of the structure in a controlled manner, with reduced uncertainties related to the actual placement and characteristics of the load. It is possible to design field tests in the early stage, ultimately selecting those scenarios that allow for the most robust verification and recognition of bridge structural specificity. The main challenge in generating such a set is to ensure its accuracy and reliability in relation to future data that are observable during the operation of the system in real conditions. This objective was achieved by updating the FE model by modifying the stiffness parameters of its individual components to ensure that the simulated strain values correspond to those observed during load tests. Using an uncalibrated FE model would introduce additional uncertainties into the procedure due to the discrepancies between the response of the actual structure and its simulations. On the other hand, relying solely on field test information, possibly extended by data obtained in the operational stage, would not be controlled and reliably labeled due to unknown weight, characteristics, and uncertain structure of real road traffic. Due to the extensiveness of this subject matter and the constraints on the length of this paper, the current study does not provide a detailed description of the FE model calibration.

**Figure 3:**
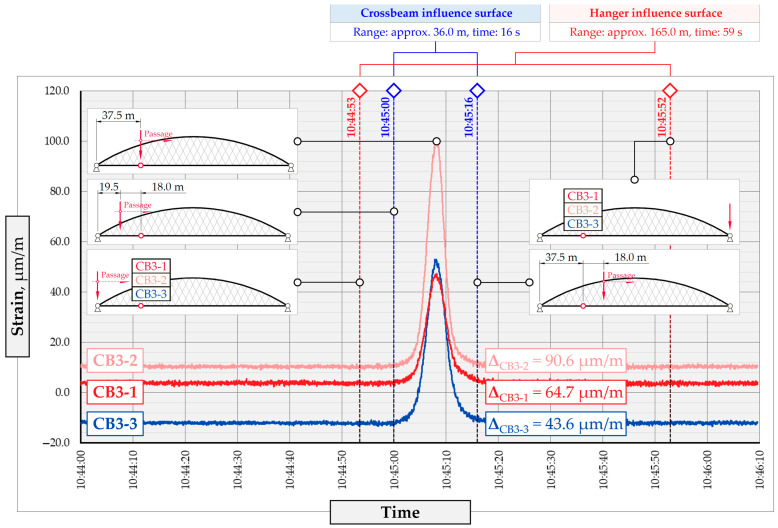
Strains in the crossbeam at gate no. 3 during the dynamic load test passage.

In addition to the static load test, the examination of the bridge structure incorporated dynamic tests using a single truck of known weight and dimensions, moving along the bridge. The tests involved passages in different positions on the width of the road, at different speeds, and in different directions, including passing through an artificial obstacle to intensify the vibrations and dynamic response of the bridge. Static and dynamic load tests allowed for the identification of the structural specificity of the bridge required for the adequate design of the SHM-based BWIM system. Figure 3 shows an exemplary change of strain measured by three sensors installed on the bottom flange of the crossbeam at the measurement gate no. 3, recorded during one of the dynamic load test passages. The test was carried out using a vehicle with a total weight of 33.0 t, moving at a constant speed of 10 ± 2 km/h. The figure illustrates the timestamps for the entry and exit of the load on the bridge, as well as the timespan that reveals the presence of the vehicle above the gate. The varied intensification of the strains is noticeable within each of the measurement points, which is directly dependent on the position of the vehicle on the width of the road. At the same time, the limited range of the influence surface for strains in the crossbeam is visible, which corresponds to an evident response only for the vehicle located in the immediate vicinity of the monitored beam. The sensors do not detect the presence of a vehicle if the distance is greater than 2 or 3 times the spacing of the intermediate crossbeams, here about 36.0 m. The limited range of the surface influence for crossbeams is an important feature of the network arch bridges compared to structures of different hanger layout. In the context of implementing the BWIM systems, it allows for reducing the impact of loads placed over a large part of the bridge, beyond the range of the influence surface. Measurement points on the crossbeams do not require additional processing or filtering to mitigate the influence of other loads in the weight detection procedure for a single vehicle within the gate. At the same time, the system in which weight detection is carried out independently by three successive gates becomes potentially useful, allowing for the averaging or cross-validation of results.

**Figure 4:**
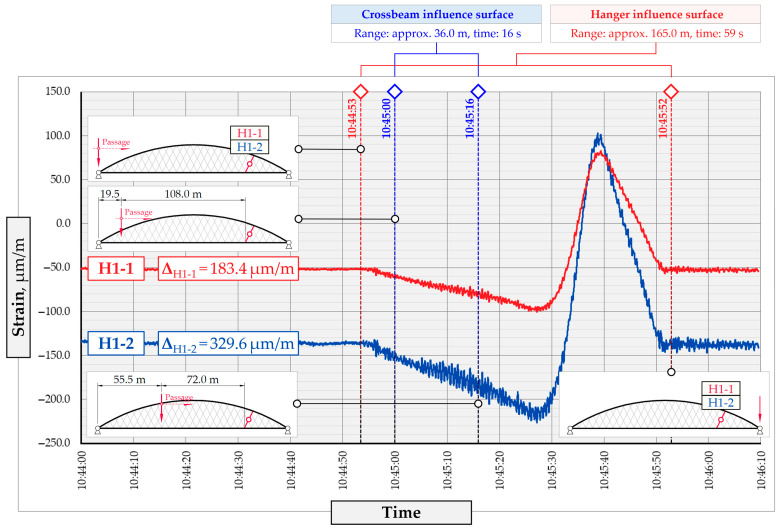
Strains in the hangers at gate no. 1 during the dynamic load test passage.

The hangers are characterized by a different shape and range of the influence surface and remain sensitive to the loads outside the gate (Figure 4). In this case, the strains in the hangers recorded during the same test indicate various scales of strain increase depending on the position of the vehicle on the bridge width. Although the timestamp of the maximum strains can also be clearly seen, corresponding to the load located right above the measurement gate, the strain values on the hangers can be affected by other vehicles passing the bridge at a given moment.

Similar characteristics of the influence range and the change in strain in time were confirmed at the other measuring points on the crossbeams, tie-beams, and hangers. The remaining tests involved different types and weights of vehicles passing the bridge in different positions related to the width of the road and at various speeds. The used vehicles, nine in total, including seven vehicles of different axle configurations, as well as an overview of the selected results obtained in the measurement gates, are given in Table 1. The other scenarios involved groups of vehicles passing the bridge in different groups and configurations. It can also be seen that relatively short vehicle-induced excitations are also not affected by loads of longer duration and significant inertia, including thermal and environmental actions. In the final implementation of the system, the differences between unloaded and loaded states are considered in the weight estimation procedure. Taking into account the specificity and structural behavior of the network arch bridge, the current research attempts to evaluate the performance of various ML algorithms for the purpose of BWIM systems, depending on the set of features considered in the input data.

### 2.3. Methods and Further Use

The objective of this study is to determine the ability of various ML algorithms to predict the position and weight of vehicles based on strain, formulating the framework of a multivariate regression. In the SHM and BWIM design stage, when not enough data may be collected prior to the operational stage, missing information can be gathered based on FE simulations. It also allows us to collect a more diverse response to a greater extent compared to real experiments conducted during the load test. The crucial requirement in this approach is to incorporate the calibrated FE model in accordance with the load test results.

The methods used in this research are as follows. Employing the updated FE model, synthetic datasets are generated. By simulating vehicles passing the bridge, each of their various weights, axis configurations, and transverse placements, the maximum strain increase is recorded in the sensors of a given gate, together with the associated strain change in the sensors assigned to the rest of the gates. The input vector includes values that are expected to be obtained from FBG sensors, with a maximum dimensionality corresponding to the number of measurement points, specifically 17 in this case. The size of the input vector is then reduced to assess the influence of the input configuration on the accuracy and reliability of the predicted output. The output values include the total weight of the vehicle and its transverse position.

Three ML models of various architectures and the number of inputs are trained and compared: linear regression (LR), random forest (RF), including its variant of the single-ensemble decision tree (DT), and neural networks (NN). A synthetic data set of 6000 samples was generated and divided into training and test sets of sizes 4800 and 1200, respectively. The input features consist of strain values, and the output includes the coordinates and weight of the vehicle. The accuracy of each regression model was evaluated using the root mean square error (RMSE) as the primary metric. The same input structure was used for all algorithms to compare their predictive accuracy. The equation is shown as follows:

(1) $$RMSE\left(X,\;h\right)=\sqrt{\frac{1}{m}\sum_{i=1}^{m}{\left(h\left(x^{\left(i\right)}\right)-y^{\left(i\right)}\right)}^{2}}$$

where:

m—number of elements in the data set;

X—matrix containing the values of all features (excluding target values) of all samples;

$x^{\left(i\right)}$—vector of feature values (excluding target values) of the *i*th sample;

$y^{\left(i\right)}$—target value of the *i*th sample;

h—model’s prediction function.

Three input configurations are tested for each of the ML regression methods. First, all 17 strain values are incorporated, assessing the accuracy of the weight and transverse placement estimation. In the next step, the reduction to 5 inputs is considered, consisting of measurement in a single gate, involving three points in the cross-beam and two points in the hangers. Finally, only 3 inputs are considered, corresponding to three measurement points in the gate-specific cross-beam. The purpose of this study is to confirm that the reduced number of inputs can still allow a reliable estimate of vehicle weight and placement, directly incorporating the specificity of the network arch bridge. The work also identifies potential risks associated with the use of synthetic data that, if no specific measures are introduced, are free of various distortion effects.

**Figure 5:**
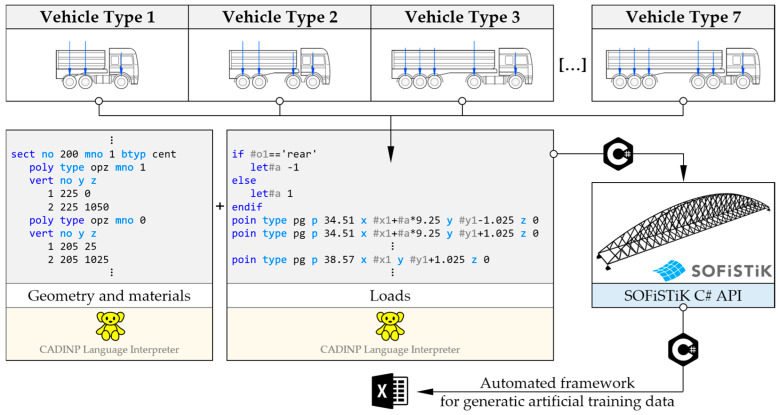
Automation of the data generation process for the purpose of training regression ML models.

To automate the generation of data sets for the training and validation of regression ML algorithms, a dedicated framework was developed, allowing for the direct use of the updated FE model (Figure 5). It was created in SOFiSTiK 2023-1 software, a commercial structural analysis environment, using text batch files with the content defined in the internal CADINP language and the SOFiSTiK API adapted to the object-oriented C# programming language. The code includes methods responsible for reading the batch files with the definition of geometry, material, and load cases, calculation, and saving the strain results to an Excel workbook. In the loadings, sets of concentrated forces corresponding to the axle arrangement of seven typical truck vehicles were provided, including those used in the load tests. The vehicles were located in different positions on the bridge and assigned different total weights, while maintaining the proportion of load distribution on the individual axles. The diversity of vehicles allows for the assessment of the universality of the ML models in predicting the total weights of vehicles with different numbers and axle spacings.

**Figure 6:**
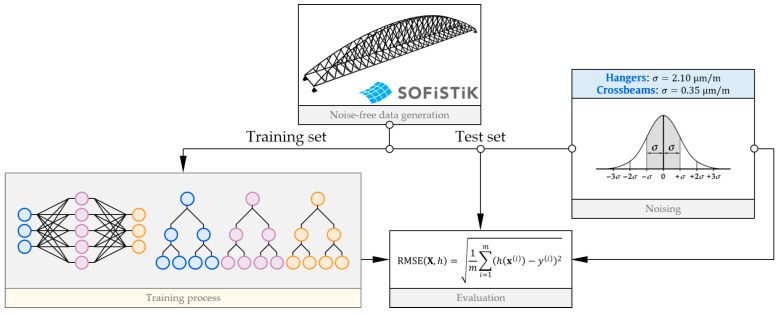
Training and performance evaluation procedure.

To estimate the influence of measurement noise on the accuracy of weight prediction, the following procedure is incorporated. Regression models are trained on the noise-free data derived from the static analysis of the calibrated FE model. Unless otherwise stated in the text, the models are evaluated by calculating the RMSE on training and test sets and, in the latter case, allowing for the assessment of generalizability on samples that were not incorporated in the training process. Both the training and test sets do not include the influence of noise at this stage. The evaluation of the ability to maintain the accuracy of the prediction is performed by calculating the RMSE on test data contaminated with standard noise, according to Figure 6. In this way, apart from the assessment of robustness to random disturbances, the overestimation of the performance on the noise-free data may potentially be highlighted.

**Figure 7:**
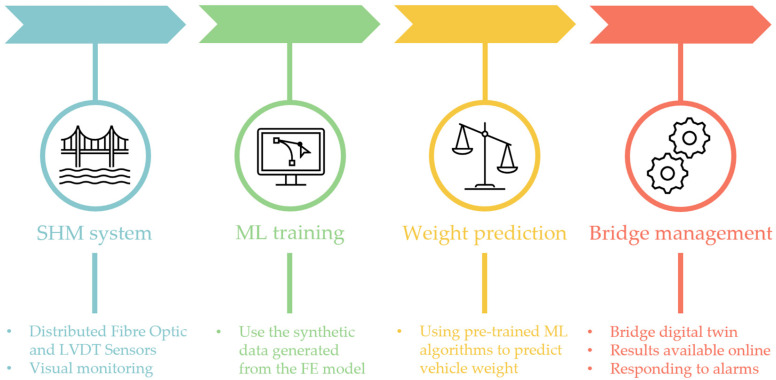
SHM system framework.

As a whole, the final implementation of the entire SHM system can be divided into the following four main stages: (1) structural monitoring, (2) training ML algorithms using the synthetic data, (3) data analysis and final predictions using ML algorithms, and (4) bridge management, as seen in Figure 7. In the first stage, fiber optic sensors record the strains in selected points of the structure in real time. Data obtained from these sensors during the load test were then used in the second stage to calibrate the FE model, based on which a set of synthetic data was generated. They served as the basis for training the ML algorithms, which were then used in the third stage for the final prediction of a vehicle’s weight and position. The ML models are automatically executed on the incoming data, and their output is continuously fed into the management layer. The last stage includes the integration of results with infrastructure management systems, such as a digital twin, a dedicated website, and a notification system for exceeding permitted threshold values to enable quick and informed operational decisions. In addition to real-time alerts, all processed data are stored for historical analysis and can be used to assess traffic load trends and structural performance over time.

**Figure 8:**
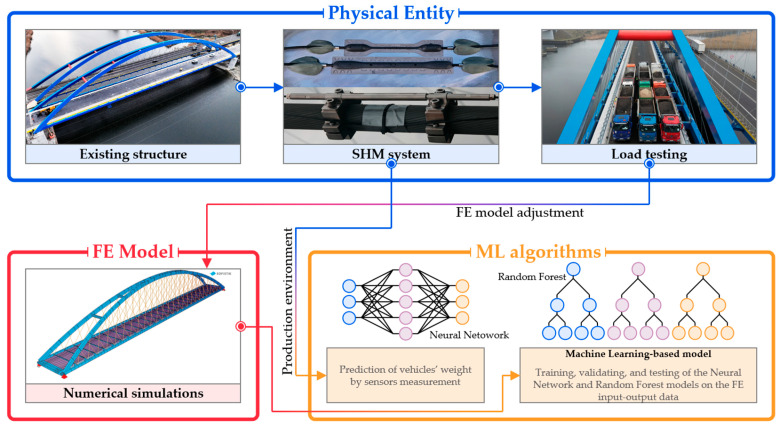
The procedure of establishing the BWIM module, including the SHM system, load testing, FE model adjustment, and ML regression algorithms.

Figure 8 presents the data flow for the purpose of the ML-based approach incorporated into the SHM system. It consists of the real structure with the installed sensors and the digital FE model that reflects its structural behavior and specifics. The FE model, once updated in the calibration process, is used to generate the training data set. In the current framework, several regression ML algorithms of various architectures and number of inputs are evaluated and compared in terms of precision in predicting vehicle weight and coordinates. The best-performance model can be utilized to establish a BWIM module in the final system, wherein continuous sensor readings are used to collect information about road traffic and load magnitude on the bridge.

## 3. Results

### 3.1. Linear Regression-Based Predictive Models

Linear regression (LR) is one of the basic regression models used to generalize the relationship between a dependent variable and a set of independent inputs by fitting a linear function to the observed data. In this research, the LR model is used as a baseline for further comparison with more sophisticated regression methods, allowing for the assessment of linearity in data obtained from linear FE models. At first, the LR predictor is trained on the strain values recorded by all sensors in the system at a specific timestamp. The performance of the regression model is analyzed by the RMSE value of the predictions on the weight and transverse position of the vehicle, Y. As seen in Figure 3 for the strains in the crossbeam, the exact timestamp that triggers the predictions, as well as the longitudinal placement of the load, X, can be clearly indicated by the peak values in the strain time series data.

**Figure 9:**
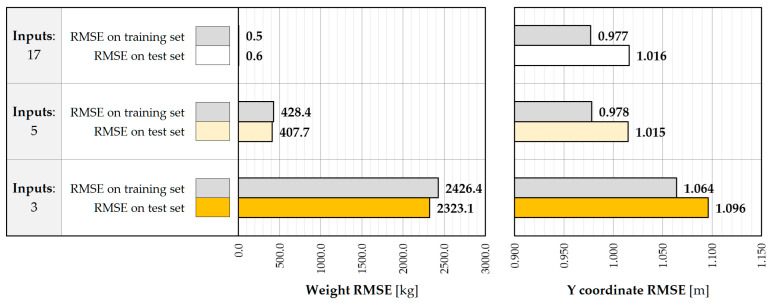
Weight and Y coordinate RMSE for LR model with 17, 5, and 3 inputs.

Figure 9 shows the performance of the LR model depending on the set of input features. Providing strain values measured in all 17 points reduces the RMSE in the weight estimation procedure to negligible values, revealing a seemingly high accuracy. However, this performance should be interpreted in a broader context, examining the influence of data structure and their quality on the generalizability of the model. The current data include strain values recorded by all sensors at the timestamp of maximum excitations at a given gate. It turns out that, at this time, the strains in the distant crossbeams oscillate around relatively small values of low variance, caused by the limited range of their influence surface. At the same time, the training data reflect the results of the linear static analysis performed on the calibrated FE model and do not include the noise that is common in real-world applications. Under these circumstances, the LR model assigns large weights to small strain values at distant crossbeams, attributing them to a significant influence on the model fit. Even a small deviation from the expected value, including that due to noise effects, causes a disproportionate change in the weight estimate and a significantly large RMSE value. Training non-regularized regression models, including LR, on the simulated data with no noise poses the risk of overestimating their performance and reducing robustness in the real-world environment. Limiting the input features to five and three inputs decreases the performance of the model, resulting in an RMSE of 407.7 kg and 2323.1 kg on the test set, respectively. The results reveal non-linear relationships between the explanatory variables and the output. At the same time, the vulnerability to noise effects is mitigated as the simplified linear function imposed on the non-linear dependencies and serves as a form of regularization. The prediction of Y coordinates is not affected by the complexity of the input features and achieves accuracies in the range between 1.016 and 1.096 m in the test set. It results from the absence of a linear relationship between the transverse position of the vehicle and the inputs as well as a very similar impact of the strain values in the closest crossbeam that are incorporated in all three models.

**Figure 10:**
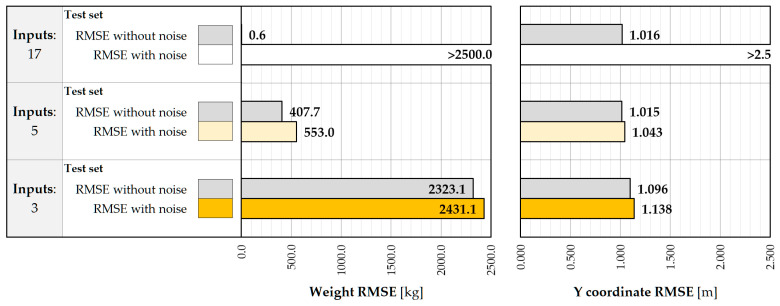
Weight and Y coordinate RMSE for LR model with 17, 5, and 3 inputs, with noise included.

The sensitivity to noise can be analyzed following the procedure mentioned in Figure 8. As can be seen in Figure 10, the accuracy of the 17-input model decreased significantly, due to the overestimated weights assigned to the strain measurements in the distant crossbeams. This effect can be mitigated by excluding the measurement points from the input features. If no additional measures are used to reduce noise effects on the input values, then the vulnerability of the five- and three-input models becomes apparent through the increase in the RMSE value of about 150 kg, despite the relatively overall low performance of LR.

The drawbacks of the LR model and its oversimplification for a reduced number of input features do not allow for achieving the required level of accuracy according to project specifications, 0.5 t. Performance in this context can be considered unsatisfactory and justifies the use of more sophisticated regression methods.

### 3.2. Random Forest-Based Predictive Models

Random forest (RF) is one of the ensemble ML algorithms used in various classification and regression tasks. For the latter, it consists of a given number of regression decision trees, each of which is independently trained on a specified group of samples from the training set. The training process consists of gradually expanding the tree by splitting the set into two nodes. Newly created nodes are progressively split into deeper subsets until a stop criterion is met, usually reaching the maximum tree depth or minimum number of samples in the node. In the current approach, the best split in the node is obtained by minimizing the average squared difference between the feature values of the samples assigned to the potential sub-nodes and their means. The splitting algorithm evaluates every possible split and selects the one that results in the greatest reduction in mean squared error in the resulting subsets. This process is repeated for given and subsequent nodes until a single element leaves at the end. A single RF consists of several decision trees, making the model more diverse and reducing the variance of the entire forest. The final prediction is calculated as the average of the outputs returned by each of the trees.

In the current study, RF models with different numbers of decision trees and different numbers of training samples in relation to the size of the whole dataset are considered and compared with the performance of the NN regression models. At first, different RF architectures are trained on the strain values recorded by all sensors in the system at a specific timestamp. The performance of the regression model is analyzed by the RMSE value of the predictions on the weight and transverse position of the vehicle, Y.

**Figure 11:**
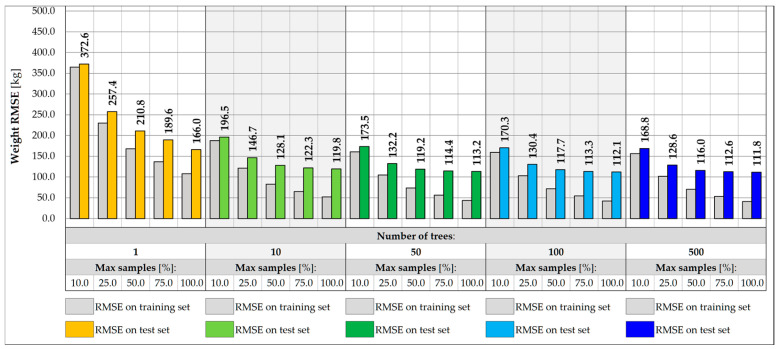
Weight RMSE for different RF architectures with 17 inputs.

**Figure 12:**
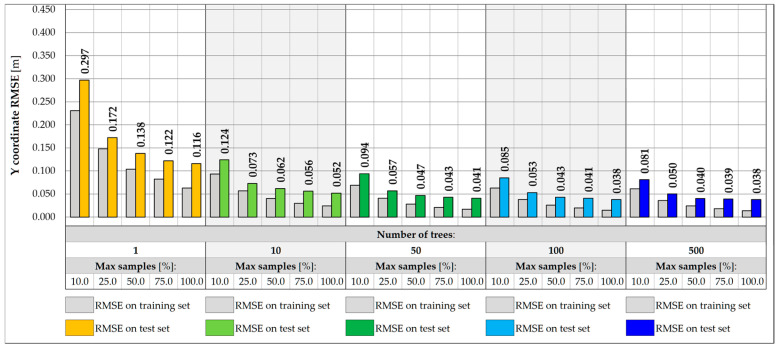
Coordinate Y RMSE for different RF architectures with 17 inputs.

Figure 11 and Figure 12 show the impact of the RF architecture on the prediction error for vehicle weight and its transverse position, Y, respectively. Five various numbers of trees are assumed, in turn 1, 10, 50, 100, and 500 trees. For each number, different relative sizes of the training subsets are analyzed, labeled as max samples, and range from 10.0 to 100.0%. The models are evaluated on training and test sets to estimate the variance and assess the ability of the model to generalize the results for inputs that did not participate in the learning process.

For single-tree architectures, the RMSE values are in the range from 166.0 to 372.6 kg for weight and 0.116 to 0.297 m for coordinate Y. The variance of a single decision tree is regularized by the introduction of sampling with replacement, which allows for the repetitive occurrence of samples in the training subset, while keeping some of the samples out of the badge. In most cases, an effect of overfitting is observed, in which the error measured on the test set is higher than the error on the training set. The problem can potentially be solved by introducing more trees or using additional regularization techniques, including reducing the size of the training subsets. The overfitting effect is reduced only for drastically reduced subsets, at least 25.0.% of the initial set size. Despite the effect, the best performance model is usually chosen based on the error measured on the test set. Following this assumption, it can be seen that the higher the number of tress and the larger the size of the subset, the higher the accuracy of the RF model. For models consisting of at least 50 trees and trained on at least 75.0% of the initial set, the accuracy measured on the test samples lies in the range from 111.8 kg to 114.4 kg for total weight and 0.038 to 0.043 m for Y estimations. It is possible to achieve an accuracy of less than 50.0 kg for the training set.

**Figure 13:**
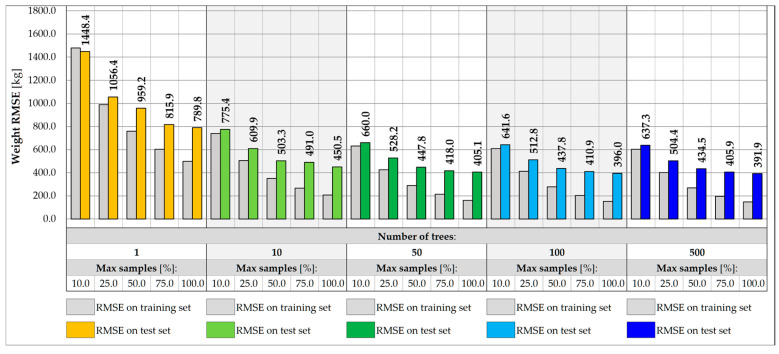
Weight RMSE for different RF architectures with 5 inputs.

**Figure 14:**
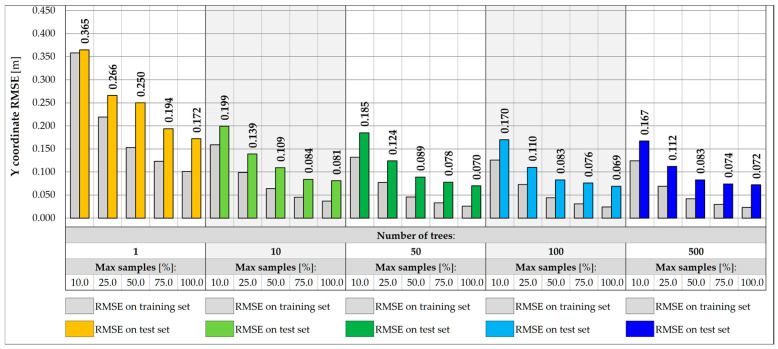
Coordinate Y RMSE for different RF architectures with 5 inputs.

Reducing the feature set by excluding the strains measured at the points outside the given measurement gate allows for limiting the potential impact of vehicles located in other parts of the bridge. In this case, a five-element input vector is considered, consisting of strain points on the crossbeams and two hangers on the left and right sides of the deck. In the example of gate no. 1, the accuracy of the weight and transverse position predictions decreases for all RF architectures considered, as shown in Figure 13 and Figure 14. For the best model identified for the entire set of features, the RMSE value measured for the test set drops from 111.8 kg to 391.9 kg, which is still satisfactory, considering the expected traffic load range estimated up to 40 t. The achievable accuracy for the prediction of the transverse position reaches 0.070 m.

**Figure 15:**
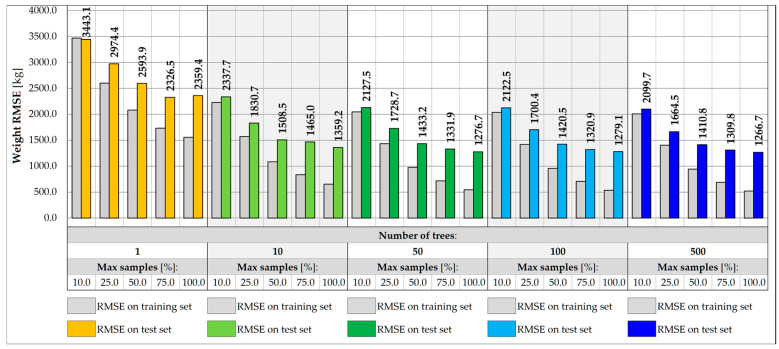
Weight RMSE for different RF architectures with 3 inputs.

**Figure 16:**
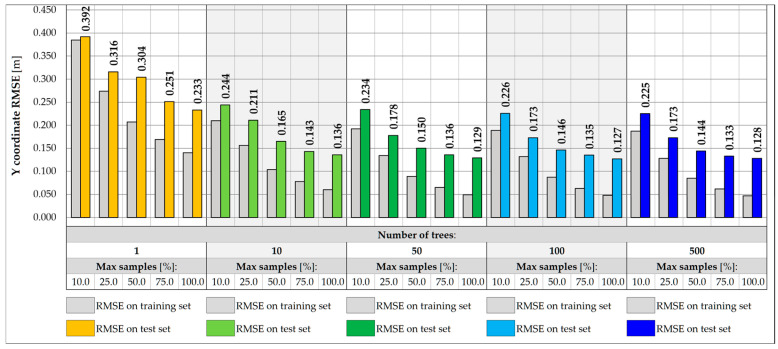
Coordinate Y RMSE for different RF architectures with 3 inputs.

Further reduction of features in the input vector may involve excluding strains in hangers while maintaining the strain reading in the measurement gate crossbeam. In this case, due to the limited range of the surface influence, the RF input values depend only on the vehicles located within the gate and its closest vicinity. This approach allows for exclusion of the use of algorithms that filter the influence of distant loads, which, however, involves further reduction of the accuracy of predicting weight and transverse position. The RMSE values measured in the training and test set for the three-input vectors for the same RF architectures are shown in Figure 15 and Figure 16. For the best model identified so far, the RMSE value measured for the test set drops to 1266.7 kg, which exceeds the required level of accuracy assumed in the specification, 0.5 t. The RMSE for the transverse position is less than 0.130 m.

It was also noted that the performance and accuracy of the RF models depend to a large extent on the size of the training set (Table 2). With 4800 samples in the training set, the learning time of a 500-tree three-input RF model was within 6 s, which is much shorter compared to the time required to train more advanced models, including neural networks. As seen in Table 2, a tenfold increase in the size of the training set allows for the reduction of the RMSE value in weight prediction to an acceptable level of 509.6 kg, with an increase in training time to 73.2 s.

The influence of noise measured by an increase in the RMSE value for the noise-contaminated test set for the best-performance RF models was analyzed. In the case of the 17-input regression, the accuracy of the weight prediction was significantly reduced, exceeding the error of 3.5 t, which does not qualify the model for further development and deployment in the real structure. The reason for this effect is again the too high significance of the strain values in the distant crossbeams. Oscillating around relatively small values of a magnitude similar to that of noise disrupts the data flow in each of the decision trees, leading to incorrect inference about the weight of the vehicle. The models trained on a reduced number of features, excluding strains in the distant crossbeams, are able to mitigate the noise impact. The increase in RMSE is 124.6 kg and 60.0 kg for models with five and three inputs, respectively.

Based on the results obtained, the RF regression models of a reduced number of inputs are close to achieving the required level of accuracy of 0.5 t, according to project specifications, for both training and test sets. However, it requires models of higher complexity, involving at greater number decision trees, at least 500, and a data set of significant size, about 48,000 samples in the reported case. Although the latter legitimates the use of updated FE models and synthetic data in the evaluation of various ML architectures prior to their final deployment, several limitations may be observed in the context of use on real data. First, the evaluation using synthetic data does not involve uncertainties that can exist in real-world applications, including noise, impact of environmental effects, diversity of traffic scenarios, and nonlinearity of structural response. Second, the RF models examined still exhibit the effect of overfitting. Although the overfitting could be addressed by introducing additional regularization, including reducing the depth of decision trees or imposing a minimum number of training samples in leaves required for the growth of each tree, it would require a further increase in dataset size to maintain the accuracy obtained. Consequently, it is desirable to develop models for which the accuracy achieved will be significantly below the required threshold, while mitigating the effect of overfitting.

### 3.3. Neural Network-Based Predictive Models

In this research, which consists of the prediction of the weight and location of the vehicles passing by the bridge, neural network (NN) models can also be used. This is an example of a regression problem, in which NNs have high accuracy and effectiveness. These models are used for complex predictions and the identification of dependencies between input and output data. Under their assumption, they constitute a network of interconnected neurons grouped into input, output, and intermediate hidden layers. All input data are transferred to the first layer of the network and then transformed into subsequent layers according to the assigned weights. The value stored in each neuron corresponds to the sum of the products of the values from neurons from the previous layer and the weights assigned to each connection. Then, it is modified by adding an additional deviation and is transformed using an activation function. In practice, the operation of NNs can be described using a matrix equation that transforms the input data. During the training phase, the NN adjusts the model weights for each connection to most closely represent the known output given the input.

**Figure 17:**
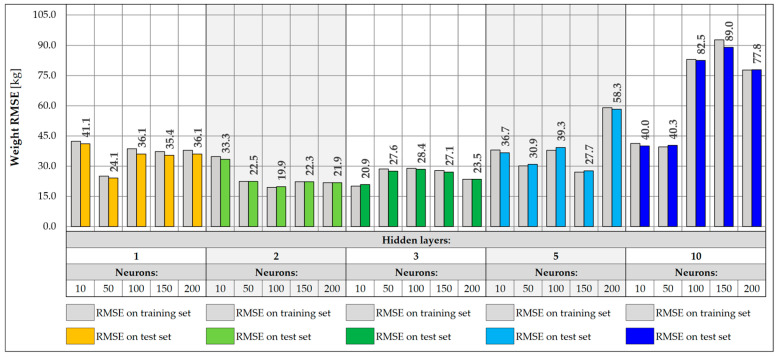
Weight RMSE for different NN architectures with 17 inputs.

**Figure 18:**
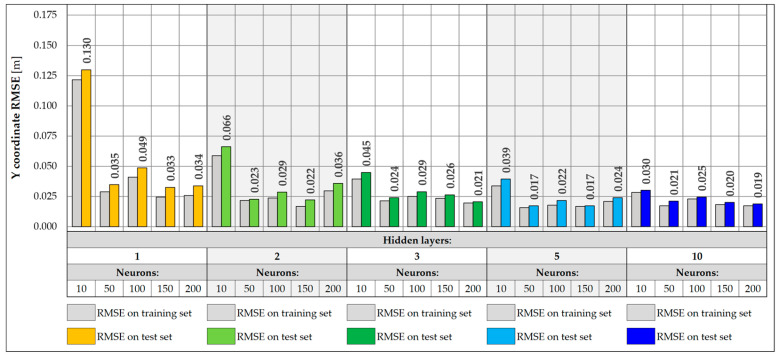
Coordinate Y RMSE for different NN architectures with 17 inputs.

When designing NNs, an important part is the selection of the appropriate architecture, in particular, the number of layers and neurons. In the presented study, a total of 25 different NN architectures were tested, including configurations with 1, 2, 3, 5, and 10 hidden layers and with 10, 50, 100, 150, and 200 neurons in each layer. The NNs were tasked with accurately predicting the location of the vehicle on the bridge in the transverse Y direction and its weight. Each of the networks was trained for a maximum of 1000 epochs, using an early stopping mechanism with the patience parameter set to 100 epochs. During NN training, the learning rate plays a very important role, defining the size of parameter changes in each iteration. Too high a value can lead to learning instability, where the model weights tend to infinity instead of stabilizing. On the other hand, too low a value leads to slow convergence and creates the risk of stopping the learning process at a local minimum. Therefore, different learning rate values were tested, and a value was chosen for which the best models were obtained. The values of 0.001, 0.005, 0.01, 0.05, and 0.1 were adopted. The loss function was the Huber function, which combines the features of the mean square error (MSE) and the mean absolute error (MAE). For small errors (below the threshold value), it has a quadratic character, making it learn faster and achieving convergence near the target values. However, above the threshold value, it takes a linear form, which makes it more resistant to outliers. The metric defining the accuracy of each architecture was the root mean square error (RMSE) calculated for the entire training and test set (Figure 17 and Figure 18).

For vehicle weight prediction, the best accuracy is achieved by an NN of moderate depth consisting of two or three hidden layers. Shallower networks with one hidden layer are not able to correctly model the relationships between data, achieving significantly higher RMSE values. There is also a noticeable decrease in accuracy for deeper networks with five or ten layers, leading to higher RMSE values. The best accuracy was achieved for an NN consisting of two hidden layers and 100 neurons, for which the RMSE is 19.9 kg. The predictions presented for 17 input data show that increasing the depth and number of network neurons does not necessarily lead to better accuracy. Choosing an architecture consisting of two or three hidden layers with at least 50 neurons is the best compromise between model accuracy and its complexity.

For prediction of the Y coordinate of a vehicle on a bridge, the best results are achieved by NNs with a depth of two to ten hidden layers. Shallow networks or those containing a smaller number of neurons were characterized by greater overfitting, resulting in higher RMSE values in the test set compared to the training set. This indicates the inability of simpler models to capture complex dependencies between data. In turn, networks with a larger number of layers did not provide further improvement in accuracy and achieved RMSE values similar to those of simpler two- or three-layer networks. The best accuracy was obtained for a network consisting of five hidden layers and 150 neurons, achieving an RMSE of 0.017 m and the lowest degree of overfitting in relation to all architectures. However, two-layer networks with 50 or more neurons achieve satisfactory results and can be a good compromise between accuracy and model complexity.

In the case described above, the input data are the strains recorded on all 17 sensors. In such a system, it may be difficult for the NN to determine the location of the vehicle in the longitudinal direction X when there are more vehicles on the bridge at the same time. This results from the specificity of the operation of the arch structure with a network suspension, which is characterized by small areas of influence for the measurement points located on the crossbeams. They cover only about two bands of crossbeams, which means that they record strains only when the vehicle is directly above a given measurement gate. Consequently, when there are more vehicles on the bridge, the NN may have difficulty accurately predicting the exact weight and location of each of them. For this reason, it was decided to use measurement gates as an effective method to determine the position of the vehicle along the length of the bridge because the results obtained are not disturbed by vehicles standing at a greater distance from a specific gate. Therefore, NN can predict the position Y and the weight of only one vehicle at a time, currently passing through the measurement gate. As a result, it can be assumed that the X coordinate of the vehicle position is equal to the position of the measuring gate along the bridge.

**Figure 19:**
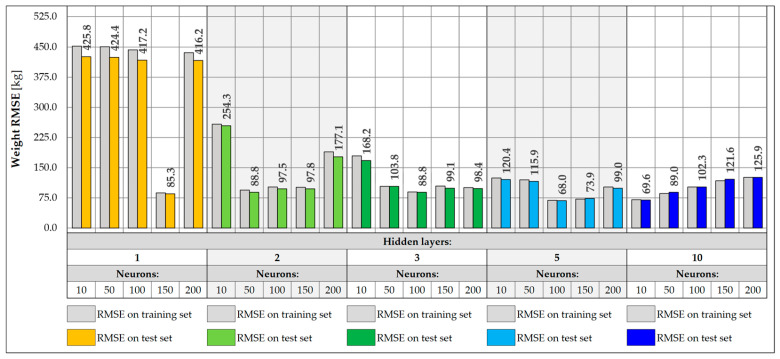
Weight RMSE for different NN architectures with 5 inputs.

**Figure 20:**
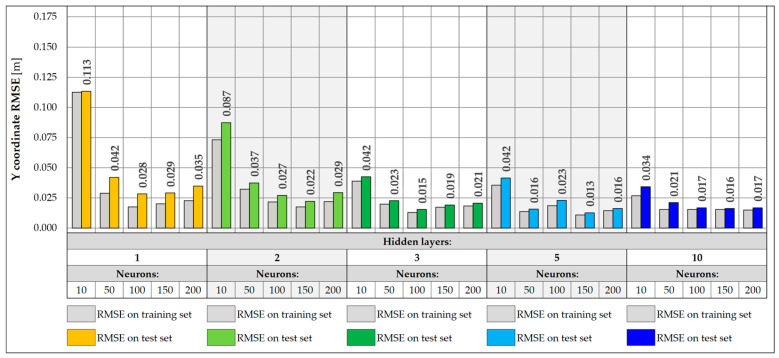
Coordinate Y RMSE for different NN architectures with 5 inputs.

NNs were trained with input data collected from a single measurement gate consisting of five sensors, located on crossbeams and hangers. The same architectures were analyzed as for 17 input data, consisting of one, two, three, five, and ten hidden layers and 10, 50, 100, 150, and 200 neurons, respectively. Each network was trained for a maximum of 1000 epochs, assuming an early stopping mechanism with a patience factor of 100 and analyzing different values of the learning rate, 0.001, 0.005, 0.01, 0.05, and 0.1, respectively, choosing the value for which the model performed best. The RMSE values were then determined for the training and test sets (Figure 19 and Figure 20).

In the case of weight prediction with five input data, networks with at least two hidden layers and 50 neurons perform very well, achieving RMSE values below 100.0 kg. On the other hand, the shallow network with one hidden layer is too simple and was unable to effectively model the appropriate relationship between data for any learning rate. These networks (except for the one with 150 neurons) stopped at a local minimum and were unable to fully carry out the learning process. Their training was stopped already around the 300th epoch using the early stopping mechanism, which indicates that they did not improve their accuracy during the subsequent epochs. There is also a slight increase in the RMSE values for more complex architectures, including 10-layer networks, which achieve RMSE values exceeding 100.0 kg. The best accuracy was achieved by the NN with five hidden layers and 100 neurons, for which the RMSE is equal to 68.0 kg.

The best accuracy in determining the vehicle Y coordinate is achieved by networks with at least three hidden layers and 50 neurons. There is also a slight improvement in accuracy compared to networks using all 17 input data. Also in this case, increasing the depth of the network does not lead to achieving better accuracy, and networks with three or five hidden layers are good solutions, which are also not complex architectures. The best accuracy was achieved by a network with five hidden layers and 150 neurons, for which the RMSE value is 0.013 m.

**Figure 21:**
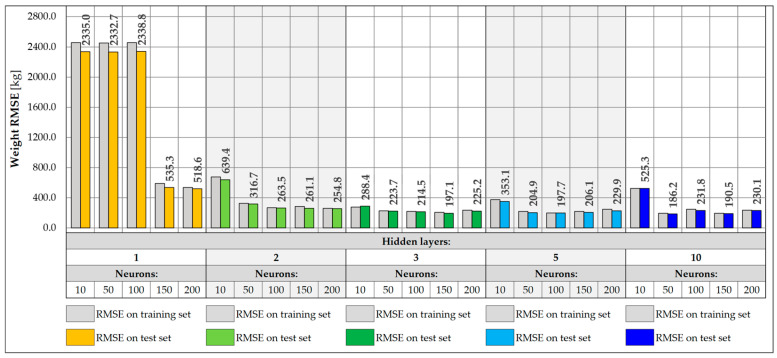
Weight RMSE for different NN architectures with 3 inputs.

**Figure 22:**
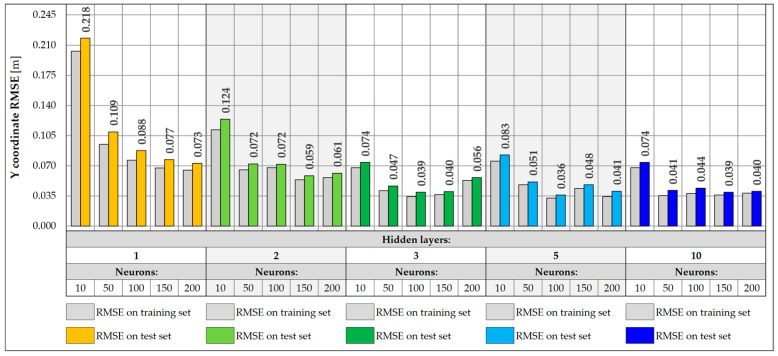
Coordinate Y RMSE for different NN architectures with 3 inputs.

The disadvantage of the models using five inputs is the consideration of strains from the hangers. Although the influence surface for the crossbeams is rather limited and covers a narrow range, for the hangers, it reaches much further along the bridge. As a result, with a larger number of vehicles, the effectiveness of these models can be disturbed by vehicles standing in front of or behind the measuring gate. Further data filtering and NN training show a greater influence of the model architecture on its accuracy. In this case, the RMSE values are larger than in the previous models, but they are not disturbed by other vehicles that are not located above the specific measuring gate. At the same time, the accuracy achieved by these networks is sufficient in the conducted study (Figure 21 and Figure 22).

In the case of vehicle weight prediction, NNs with at least two hidden layers and 50 neurons performed very well, being able to make predictions with an accuracy of about 200 kg, which is sufficient for vehicles weighing up to 40 t. Shallow networks with one hidden layer and a small number of neurons were too simple and were unable to correctly complete the learning process, stopping each time at a local minimum, while checking different variants of the learning rate. Three-layer networks provide a good compromise between accuracy and model complexity and can be successfully used in vehicle weight prediction. On the other hand, the best accuracy was achieved by an NN with 10 hidden layers and 50 neurons, for which the RMSE value is 186.2 kg.

The RMSE for predicting the Y coordinate of the vehicle’s position decreases with the increase in the number of neurons in the network layers. However, for NNs with at least three hidden layers and 50 neurons, they already reach similar values that constitute good solutions, with not necessarily complicated architectures. On the other hand, shallower networks, especially those with one hidden layer, do not generalize new data well and perform much worse, achieving about twice as high RMSE values and being characterized by slight model overfitting. The best accuracy is characterized by a network with five hidden layers and 100 neurons, which reached an RMSE value of 0.036 m.

For each of the feature sets analyzed, the NN-based regression model is able to produce results with required accuracy, 0.5 t. However, by examining the performance of the models on a test set containing noise effects, a significant increase in the RMSE value for the 17-input vector is observed. Similarly to LR and RF regression models, the sensitivity to noise can be mitigated by incorporating models with a reduced number of features where the distant crossbeams are excluded from input features in a specific gate. In their case, the increase in RMSE values does not exceed 100.0 kg.

### 3.4. Model Accuracy

To check the accuracy of the ML algorithms, regression models were implemented. Perfect fitting occurs when the error is equal to 0.0, meaning the regression model takes the form x=y, where *y* is defined by the following Equation (2):

(2) $$y=\beta_{0}+\beta_{1}x+\xi$$

where:

$\beta_{0}$, $\beta_{1}$—structural parameters of the function;

ξ—random component.

In this regression model, $\beta_{1}$ is a coefficient indicating the deviation of the predicted results from the measurement. The average difference (standard error of estimate) between measured and predicted values is the simulated error, where σ is the variance of the model residual.

**Figure 23:**
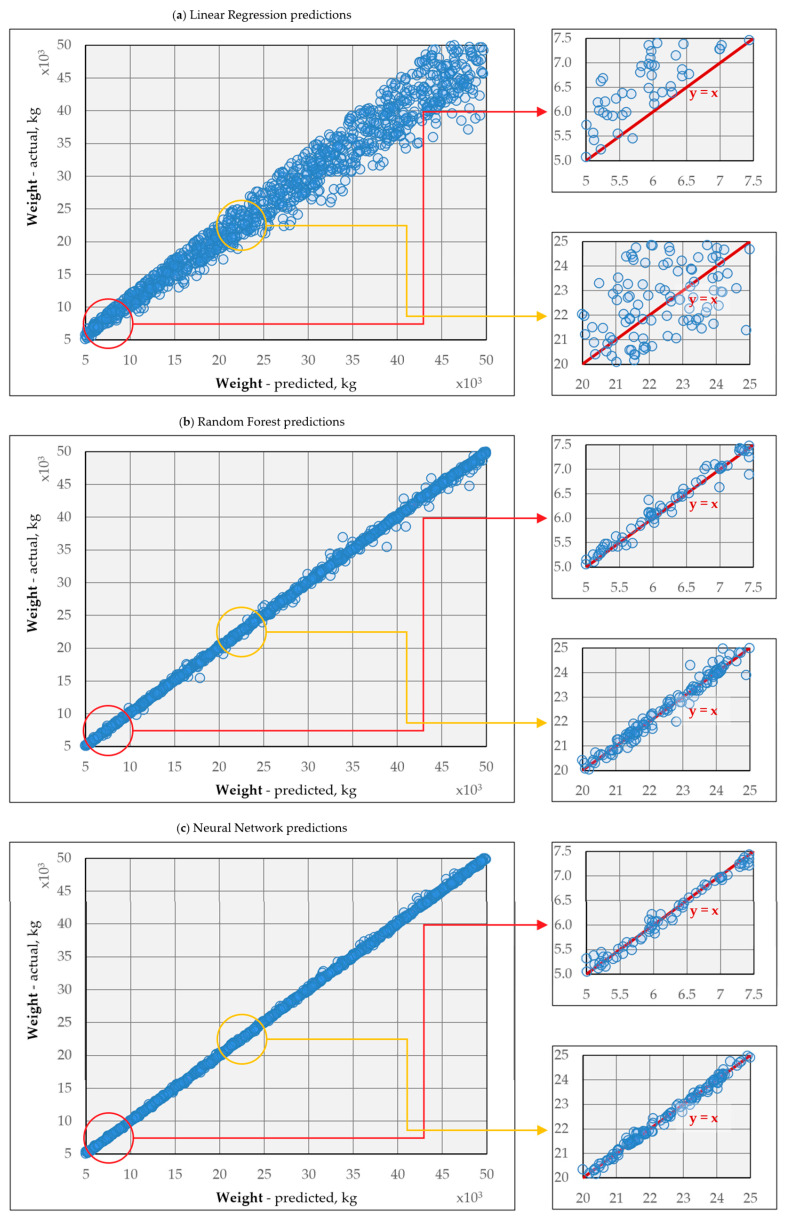
Model accuracy for predicting vehicle weight based on 3-input vector: (**a**) neural network regression model, (**b**) random forest regression model, (**c**) linear regression model.

Therefore, σ is a benchmark of model fit, based on the model residual, which means the discrepancies between the actual values of the dependent variable in the sample and the value of the convergent dependent variable calculated on the basis of the model. Therefore, the goal is to achieve a situation where the variance value tends to 0: σ→0. Figure 23 presents the regression models obtained from the best performing models predicting the vehicle weight based on three input data. These models are a linear regression (Figure 23a), an RF consisting of 100 trees with the tree size built by sampling with replacement and the size equal to the initial set (Figure 23b), and an NN with 10 hidden layers and 50 neurons per layer (Figure 23c).

The models presented in Table 3 were trained on data without noise. The best predictions of weight and Y position were achieved when using all sensors, i.e., 17 input data sets. The impact of noise was determined by introducing noise into the test set, not the training set, which allowed for an estimation of any potential degradation in the accuracy of the algorithms. This degradation occurred in each model, but for RF and NN, it did not exceed 124 kg and 100 kg, respectively, with appropriately selected architectures. It was also noted that unregularized models, such as LR, for which the RMSE value increased by more than 2.5 t, are the most sensitive to noise. Therefore, special care should be taken when training models on synthetic data, which do not incorporate various distortions, including noise. However, it is possible to train algorithms on noisy data or apply input filtering procedures to mitigate noise.

## 4. Conclusions

This article presents the implementation of the BWIM framework based on fiber optic sensors and FBG technology. Its design integrates the data obtained from the SHM system and ML algorithms to predict the total weight and position of vehicles on a network arch bridge. To generate a training set, a load test of the bridge was carried out, during which strains measured by the fiber optic sensors were recorded. The strain measurement points were placed at key points of the structure to ensure an accurate representation of its response and operation. Based on these load tests, the FE model updating procedure was performed, enabling training set generation and assessment of the specificity of the bridge structure on the final operation of the BWIM system.

The analysis showed that reducing the number of sensors and focusing on specific measurement gates eliminated the influence of other loads on the bridge due to limited influence surfaces. Each sensor records the impact of a vehicle located in close proximity to the measurement gate, and its indications are not disturbed by other loads and vehicles located at a further distance.

To predict the total weight and position of vehicles on the bridge, the accuracy achieved for the linear regression (LR), random forest (RF), and neural network (NN) models was compared with various hyperparameters and architectures. In this study, after reducing the number of sensors and training the model on three input features, the accuracy achieved for the RF with an architecture consisting of 500 trees was about 1.3 t and 0.5 t for 4800 samples and 48,000 samples in the training set, respectively. Increasing the number of decision trees, as well as increasing the size of the subsets used to train individual trees, improved performance, but led to overfitting.

To achieve better accuracy and reduce the effect of overfitting, NN models were introduced into the system with various architectures, including the number of layers and neurons. It was found that a deeper and more complex NN did not necessarily give better results, and often, good correspondences could be obtained for a moderate depth consisting of 2–3 hidden layers and about 50–100 neurons. This solution provides a good compromise between the accuracy of the model and its complexity, which significantly affects the computation time. Shallower networks consisting of only one hidden layer are often too simple to model complex dependencies in the data and are also susceptible to overfitting. In addition, a significant influence of the learning rate on the learning process of NN was identified. Therefore, it was decided to analyze different values for each of the architectures. Similarly to RF, the best results were achieved when 17 sensors were used as input data. The gradual reduction of features slightly worsened the accuracy of the model, finally achieving about 200 kg for three input data. Despite the higher error value compared to the training models on the full set of input features, the accuracy was sufficient from the point of view of the target application, in which the expected load intensity reached the order of 30–40 t. When the number of features in the input data is reduced, the influence of other vehicles located outside the area of influence of the crossbeam gates is eliminated.

However, the methodology presented has certain limitations that require further research. Fiber optic sensors installed to the structural elements and hangers did not allow the axles to be distinguished from passing vehicles, making it impossible to determine the exact type of vehicle. Furthermore, the presented research focused on the occurrence of one vehicle within the measurement gate. The surface of influence for which the sensors register the vehicle is approximately twice the crossbeam spacing, i.e., approximately 12 m. A situation in which two or more vehicles are simultaneously in this area of the bridge, if it is relatively rare, requires appropriate preparation and calibration.

Future work includes generation of a digital twin (DT) based on sensors and the SHM system. Due to the prediction of vehicle weight and location determined on the basis of a limited number of sensors reading, it is possible to simulate load effects and compare them between the FE model and the remaining sensors. A system constructed in this way provides the possibility of full automation of the structural monitoring and an anomaly detection system, in which the basic elements, in addition to the ML algorithms, are installed fiber optic sensors.

## Figures and Tables

**Table 1:** Overview of the vehicle and strain development during dynamic load tests.

Vehicle Type, Dimensions, and Weight	Crossbeam Strains	Hanger Strains
**Vehicle 1** 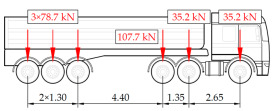 Type: **1**—Total weight: 41.4 t	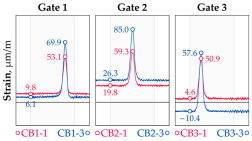	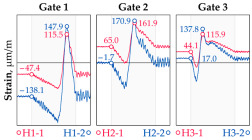
**Vehicle 2** 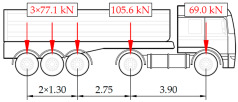 Type: **2**—Total weight: 40.6 t	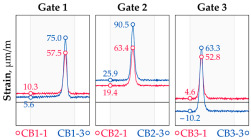	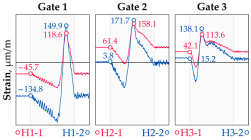
**Vehicle 3** 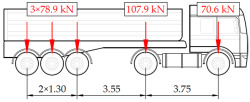 Type: **3**—Total weight: 41.5 t	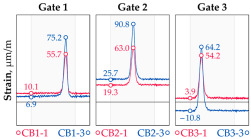	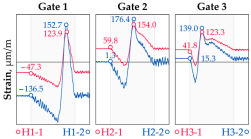
**Vehicle 4** 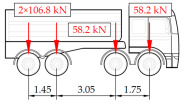 Type: **4**—Total weight: 33.0 t	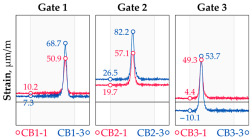	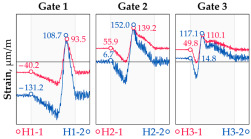
**Vehicle 5** 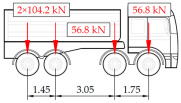 Type: **4**—Total weight: 32.2 t	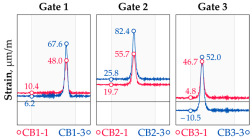	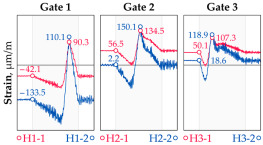
**Vehicle 6** 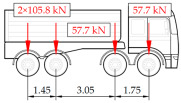 Type: **4**—Total weight: 32.7 t	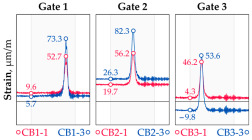	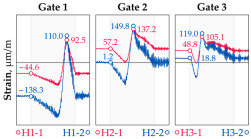
**Vehicle 7** 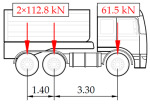 Type: **5**—Total weight: 28.7 t	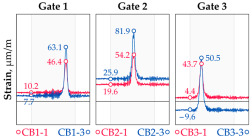	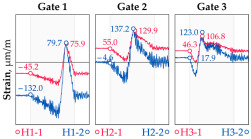
**Vehicle 8** 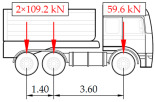 Type: **6**—Total weight: 27.8 t	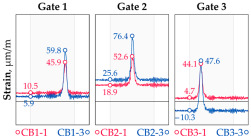	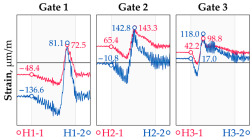
**Vehicle 9** 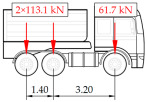 Type: **7**—Total weight: 28.8 t	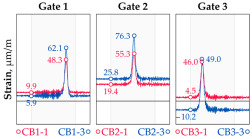	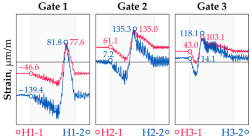

Single-vehicle dynamic tests included only, speed: 80 km/h ± 2 km/h. Transverse positions along the right lane, Szczecin → Wolin, 7.4 m ± 0.5 m from the tie-beam TB2 acc. to Figure 1.

**Table 2:** Comparison of accuracy and learning time for different sizes of the training set.

**Random Forest Regression**: Total weight estimation number of trees: 500, max samples: 100.0%			
**Training Set Size**	**Training Time ^1^ [s]**	**RMSE [kg]**	
		**Training Set**	**Test Set**
4800	5.5	518.1	1266.7
12,000	14.4	356.8	883.4
24,000	33.1	271.9	674.1
48,000	73.2	197.7	509.6

^1^ Personal computer, processor: Intel Core i9-14900HX, 32.0 GB RAM, GPU 12 GB.

**Table 3:** Comparison of accuracy for different ML models.

Number of Inputs	Predictions	Linear Regression (LR)	Random Forest (RF)	Neural Network (NN)
17 Inputs	Weight [kg]	0.600	111.8	19.9
	Y Coordinate [m]	1.016	0.038	0.017
5 Inputs	Weight [kg]	407.7	391.9	68.0
	Y Coordinate [m]	1.015	0.069	0.013
3 Inputs	Weight [kg]	2323.1	1266.7	186.2
	Y Coordinate [m]	1.096	0.127	0.036

## Data Availability

Data are available upon request from the authors.
